# Point-of-Care HIV Testing and Linkage in an Urban Cohort in the Southern US

**DOI:** 10.1155/2013/789413

**Published:** 2013-09-17

**Authors:** Anne Zinski, Sarah M. Dougherty, Ashutosh Tamhane, Kelly L. Ross-Davis, James L. Raper

**Affiliations:** ^1^University of Alabama at Birmingham School of Medicine, Division of Infectious Diseases, 845 19th Street South, Bevill Biomedical Research Building 206B, Birmingham, AL 35294-2170, USA; ^2^University of Alabama at Birmingham, School of Public Health, Department of Biostatistics, 1665 University Boulevard, RPHB 514-A, Birmingham, AL 35294, USA; ^3^University of Alabama at Birmingham, School of Medicine, Division of Infectious Diseases, 908 20th Street South, CCB 188, Birmingham, AL 35294-2050, USA; ^4^University of Alabama at Birmingham, School of Medicine, Division of Infectious Diseases, 908 20th Street South, CCB 245, Birmingham, AL 35294-2050, USA

## Abstract

The Southern states experience the highest rates of HIV and AIDS in the US, and point-of-care (POC) testing outside of primary care may contribute to status awareness in medically underserved populations in this region. To evaluate POC screening and linkage to care at an urban south site, analyses were performed on a dataset of 3,651 individuals from an integrated rapid-result HIV testing and linkage program to describe this test-seeking cohort and determine trends associated with screening, results, and linkage to care. Four percent of the population had positive results. We observed significant differences by test result for age, race and gender, reported risk behaviors, test location, and motivation for screening. The overall linkage rate was 86%, and we found significant differences for clients who were linked to HIV care versus persons whose linkage could not be confirmed with respect to race and gender, location, and motivation. The linkage rate for POC testing that included a comprehensive intake visit and colocated primary care services for in-state residents was 97%. Additional research on integrated POC screening and linkage methodologies that provide intake services at time of testing is essential for increasing status awareness and improving linkage to HIV care in the US.

## 1. Introduction

Prevention and treatment of Human Immunodeficiency Virus (HIV) infection have evolved through important advances in the field, and the number of new HIV infections in the United States has steadied near 50,000 cases annually [1, 2]. However one-fifth of adults and up to one half of HIV-infected young persons in the United States (US) are not aware of their positive status [3–9]. Considering these statistics, the Centers for Disease Control and Prevention (CDC) released revised recommendations for routine HIV testing during medical visits in US primary care settings [3]; but hurdles to healthcare-based HIV testing still exist at policy, organizational, and individual levels in the Unites States and abroad [10–15]. The recommendations for widespread implementation of standardized opt-out testing in US primary care settings were revised in 2011. Draft revisions in 2013 [16, 17] may address challenges associated with inadequate healthcare, targeted screening, and inaccuracies of individual risk perception [18–20]. As approximately 20% of adults with at least one prior HIV test report a recent (within last 12 months) test in venues outside of primary care offices [14], determining the factors associated with point-of-care (POC) test seeking is an important tactic for expanding serostatus awareness, particularly for medically underserved persons and those who do not have regular contact with the healthcare system. For these Americans, access to rapid, noninvasive HIV screening with expeditious result delivery and immediate linkage to care outside of opt-out medical settings is a distinct and invaluable opportunity to enter the HIV care continuum. 

Following the 2001 issuance of *The Serostatus Approach to Fighting the Epidemic *(SAFE) initiative, which highlighted individual HIV status awareness as a catalyst for reducing transmission [21], innovative HIV screening strategies that emphasized broader availability of tests and test results became a public health priority. In 2004, the US Food and Drug Administration (FDA) approved the first POC HIV 1/2 antibody test, which received a Clinical Laboratory Improvement Amendments (CLIA) waiver for oral fluid specimen use outside of phlebotomy laboratories [22, 23]. Though limited in detection of acutely acquired HIV infection, noninvasive POC methodology established increased screening acceptance, particularly among young and lower risk persons [10, 24]. Rapid testing technology also allowed for merged testing and linkage to care services, as trained HIV test counselors could deliver results with individualized linkage, along with pre- and posttest counseling, during one comprehensive visit. This technological advance eliminated multisession presentation to a medical facility with return for separate results, posttest counseling, and referral and linkage visits, which would streamline HIV care services for transient and hard to reach persons.

 Thorough and swift pre- and posttest counseling at POC, including linkage or clinic liaison services for persons who test HIV positive, may be a crucial component in the National HIV/AIDS Strategy (NHAS) plan to increase serostatus awareness to 90% by 2015 [25]. Evidence supports that persons aware of their HIV infection are less likely to transmit to uninfected persons, and that learning one's positive status may prompt initiation of risk reduction behaviors, including disclosure to primary partners [21, 26–29]. Additionally, as serostatus awareness is the first step in scientifically proven HIV treatment and prevention strategies, using either preexposure prophylaxis (PrEP), treatment as prevention, or antiretroviral therapy to improve clinical outcomes, POC serostatus awareness campaigns that aim to link persons to timely treatment play a pivotal role for the test-and-treat initiative at the commencement of the HIV treatment cascade [30, 31].

The National HIV/AIDS Strategy goal of improving timely linkage to care for newly diagnosed individuals from 65% to 85% motivated researchers and healthcare professionals to develop enhanced tactics to increase early linkage [25]. Recent literature asserts that achieving these goals can increase life expectancy and may prove to be cost effective [32]. In addition to recommendations for surveillance monitoring, initiation of early treatment, and treatment as prevention tactics, POC screening programs that emphasize results counseling and appropriate linkage strategies, while addressing factors that delay linkage to care, may improve HIV outcomes [33, 34]. 

In this study, we document successes and challenges of an integrated POC screening and linkage program in an urban setting in the Southern Unites States. In addition, we describe the HIV test-seeking cohort (years 2007–2012) according to test result and yearly trends. Further, we examine how clients who test positive are linked to primary care at the point of testing.

## 2. Materials and Methods

 The current investigation utilizes data from the University of Alabama at Birmingham 1917 Clinic HIV testing program (years 2007–2012) [35], which provides free POC testing using the OraQuick ADVANCE Rapid HIV-1/2 Antibody Test, which has 99.3% sensitivity and 99.8% specificity [22]. These tests were performed either within the context of a medical setting or at a community-based event. Testing services were initially offered by appointment and, with subsequent expanded staffing, on a walk-in basis. Based on the SAFE initiative constructs, POC testing was originally offered in this Deep South city as an ancillary service targeted toward sexual partners of HIV-infected patients attending the 1917 Clinic and was subsequently expanded to include no-cost screening for any test-seeking community member [21]. Community-based locations for the 1917 Clinic-sponsored testing during the study period included university and other educational settings, health fairs, faith-based organizations, and rehabilitation centers. Outreach sites were commonly selected at the request of community leaders, based on evidence of need according to public health data, or in conjunction with a student or faith-based organization impacted by HIV, including African-American and LGBT student groups and congregations. Nested within a university based academic medical center with numerous healthcare personnel and a preexisting sexual health peer educator and internship program, the HIV testing and linkage team for this project expanded to include more than 150 trained staff and student volunteers. 

OraQuick POC rapid HIV Tests were performed using a protocol based on the Health-Belief Model (HBM) by Hochbaum and Rosenstock (1958, 1960), a well-recognized conceptual framework for health-related interventions [36]. The HBM based risk assessment interview focused on perceived risk, benefits and barriers, enabling factors, cues to action, and self-efficacy and was created and merged with CDC and state of Alabama HIV serology templates to accommodate state reporting guidelines. Trained counselors used this standard form to describe HIV transmission, review Alabama reporting requirements for positive results, and collect pertinent data elements. Demographics, HIV and STI screening and risk history, and partner characteristics were included in each interview. Counselors used the 20-minute time period during which test results were processed for conveying tailored messages geared toward self-efficacy and discussion of risk reduction. 

OraQuick Volunteer Testing Counselors were trained to provide confidential and compassionate HIV testing, counseling, and linkage services within the context of a nonjudgmental environment. The objectives were to promote self-awareness of risk-taking behavior, decrease risk of HIV infection by addressing identified barriers and benefits for modifying behavior, and provide linkage to care for persons testing HIV positive. For this investigation, linkage was defined as confirmed attendance at one or more HIV primary care visits during the 12-month period after receiving positive results.

 The colocation of POC testing and linkage services within a Ryan White HIV clinic may have helped to alleviate barriers to care for those who received a preliminary positive result with OraQuick Rapid technology. While not all clients qualified for care at the 1917 Clinic due to insurance limitations, service area restrictions, or out-of-state residence, the goal of the POC testing team was to facilitate a primary care linkage visit within 90 days of a positive test. Consistent with the NHAS to link positive persons to medical care at time of diagnosis [25], the team made every attempt to establish a first intake appointment as part of same-day posttest counseling. Clients who decided to enroll in care at the 1917 Clinic were immediately scheduled for a linkage visit with a Project CONNECT (Client-Oriented New Patient Navigation to Encourage Connection to Treatment) staff member or case manager [37, 38]. In the alternative, clients who tested positive who elected to receive care elsewhere were followed by testing staff and their county health department until they obtained a confirmatory test and first medical appointment at another HIV clinic or by private physician. All test seekers were provided with direct access to a member of the 1917 Clinic Prevention Education staff as needed during the linkage process. For those who encountered barriers to linking to care, the 1917 Clinic testing team worked in partnership with the local health department, state peer mentors, and in-house staff to facilitate linkage and maintain contact over a 12-month period.

Our team evaluated a database of 4,397 interview responses from persons at least of 14 years of age who presented for a rapid POC HIV test over a six-year period, from January 1, 2007, to December 31, 2012. Of these, 448 clients had more than one HIV test during the study period; only the initial test was used in this analysis, yielding 3,651 unique client interviews. As clients were able to refuse any question, results are reported using a denominator of persons who were willing to provide answers to interviewers for that item.

 The sample had a total of 148 (4%) positive results; three of these were regarded as initial false negatives (though negative at first contact interview, a subsequent test was positive within 90 days of the first test result) and were excluded for subsequent analysis. Thus, linkage to care data were included for 145 individuals who had at least ninety days of follow up since a positive test result. The study protocol for this investigation was approved by the UAB Institutional Review Board.

### 2.1. Statistical Analysis

 In addition to reporting overall demographics of the population, clients were grouped by HIV test result into positive or negative for descriptive reporting; sample size was 3,651. Continuous variables were described as median with range due to skewed distribution. Categorical variables were reported using frequencies with percentages and compared using Pearson's chi-square test or Fisher's exact test. Statistical significance was set at 0.05 (two-tailed test). Data were analyzed using SAS statistical software (version 9.3; Cary, NC) and Microsoft Excel (for figures).

## 3. Results and Discussion

### 3.1. Results

Table 1 shows the POC test results by year for the 3,651 included client interviews, with a positivity rate of 3–5% each, and 4% of the total sample testing positive. Demographic characteristics of the overall population are shown in Table 2. Median client age was 27 years (range 14 to 79 years), with 58% under 30 years of age; median age of HIV positive clients was 29 years, while that of HIV negative clients was 27 years (*P* = 0.003). Gender distribution was nearly equal. The majority was Black/African American (Black/AA). Nearly all clients were residents of Alabama. 

**Figure 1:**
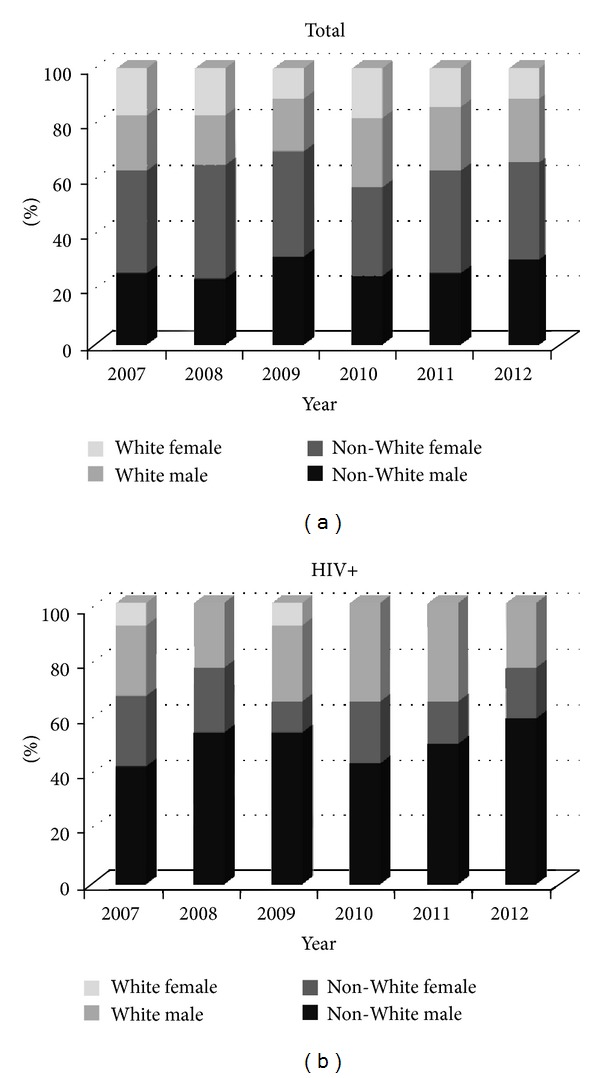
Race-Gender Distribution by year, AL, 2007–2012.

A race/gender variable was created so that interview responses for those who reported a race other than Black/AA or White were not excluded from the analysis. As the literature has described an increase in new infections among Black/AA and minority males domestically, the race/gender variable was grouped (White and non-White) to indicate whether local sample was reflective of this trend [39–43]. As illustrated in Figure 1, non-White males accounted for approximately one-quarter to one-third of the testing population each year, but were overrepresented in the positive population across years. While non-White males represented half of the positive results, non-White females accounted for less than one-fifth of the positive results. White males represented an additional 28% of the positive sample. Reported characteristics of the population were grouped according to test result. Forty-seven percent (*N* = 1, 723) of the overall population was tested at an outreach event; case finding at outreach events was less than 1%. While approximately two-thirds of the negative sample reported a previous HIV test at another location, 89% of those who tested positive reported a previous negative result, with 41% of positive clients reporting an HIV test in the previous six months (not shown).

 While 16% of all clients reported having ever been tested and diagnosed with another sexually transmitted infection (STI), nearly one-third of those who tested positive reported a history of STI (Table 3). Men who have sex with men (MSM) represented 58% of the positive population. For clients who discussed condom utilization, significant (*P* = 0.001) differences were observed, as 25% of negative and 15% of persons who tested positive reported consistent condom use (Table 3). For persons who reported any condom use, 44% of negative and 60% of positive clients reported condom utilization less than 100% of the time during vaginal or anal intercourse.

 Among persons who responded to items about partner characteristics, 27% of persons who tested positive versus 38% who tested negative reported none or a single partner (Table 4). HIV positive persons were significantly more likely to have 10 or more partners than HIV negative persons (*P* = 0.01). Partner status was also significant, as 40% of the positive clients had a known HIV positive partner as compared to 13% of the negative clients (<0.001); overall 16% were unsure of their partner's HIV status (HIV positive = 21% versus HIV negative = 16%) (Table 4).

 Fourteen percent of clients who tested positive were motivated to be tested by a self-reported high risk event or behavior (Table 4). Forty-two percent of those who were positive listed their motivation as planned or routine serostatus update. One-third of those who tested positive were motivated by a specific referral for HIV testing as compared to 10% in HIV negative clients (*P* < 0.001).

**Figure 2:**
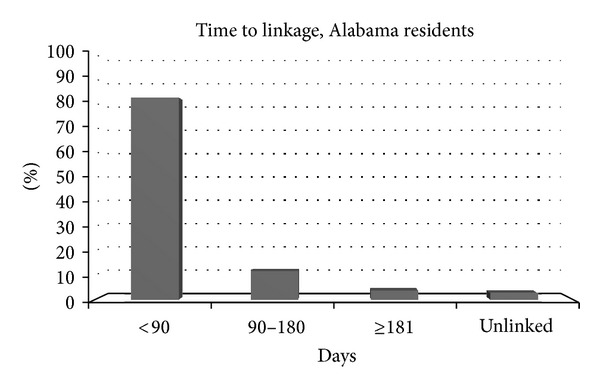
Time to Linkage in days for HIV+ Alabama Residents who participated in an integrated linkage program, AL, 2007–2012.

Of the 145 persons who tested positive at POC and were eligible for linkage follow-up, 124 (86%) had confirmed linkages to care, while the remaining 21 could not be confirmed as having attended a primary care visit in the 12 months following the positive result (unknown/unconfirmed). Of the 145 HIV positive persons, 115 elected to receive care at the 1917 Clinic and were immediately referred to Project CONNECT intake staff for a linkage visit. Time from positive test date to the arrived CONNECT intake visit was a median of five days (range = 0–378 days). Of the 115 new clients who completed a CONNECT intake, 97% (*n* = 112) arrived for a primary care visit at the 1917 Clinic. Median time from positive test to first arrived primary care appointment was 41 days, with 83% arriving for a first primary care visit within 90 days and an additional 13% arriving within 180 days of first positive test (Figure 2). In addition to these 112 clients, 12 clients confirmed that they had accessed HIV primary care at another medical facility. Thus, 124 (86%) of the 145 HIV positive results were classified as confirmed linkage to care. Of the 124 clients linked to care, most were residents of Alabama (Table 5). 

Significant differences were found between the linked to care and “unknown/unconfirmed” linkage groups with regard to gender and race-gender (Table 5). Being tested at an outreach event was significantly associated with “unknown/unconfirmed” linkage to care (*P* = 0.01) (Table 6). We did not find significant differences between the linked to care and “unknown/unconfirmed” groups with regard to previous HIV testing, condom utilization, or risk factors such as history of STI, MSM, illicit drug, or alcohol use (Table 6). Similarly, no significant differences were observed with regard to number of sexual partners or other partner-related characteristics (Table 7). Also, motivation for testing and referral source did not differ significantly between the two groups, with the exception that 38% of the linked clients were motivated by a specific referral for testing as compared to 11% in the “unknown/unconfirmed” group (*P* = 0.03) (Table 7).

### 3.2. Discussion

 Overall, more females than males were present in the testing sample (52% versus 48%), and higher numbers of Black/AA participants than White (59% versus 36%), which is representative of the surrounding Birmingham, AL, metropolitan area and local census figures [44]. Overall trends showed a positivity rate of 3–5% across all years and high rates of reported history of HIV testing across groups.

Non-White males accounted for one-quarter of overall test-seeking clients and bore the majority of positive results, as shown in Figure 1, and more than half of persons who tested positive were MSM. This is consistent with the current HIV literature that reflects an overrepresentation of positive MSM and minority males, as well as disparities, discrimination, and social inequalities that may contribute to disproportionately high rates of HIV/AIDS in the Black/AA population in the South [40, 45, 46]. Additionally, as only 14% of persons who tested positive in this sample were motivated for testing by perceived high risk behavior, and timely utilization of HIV testing services among MSM has been observed in other studies, it may be valuable to expand high impact prevention strategies for this population by providing individual and couples testing services for MSM as well as offering PrEP to persons with higher risk behaviors [47–49].

 Self-reported motivation and referral source for testing were of particular interest and, as with previous investigations of this population, convenience and individual “routine” status updates were chief motivators for testing in this population overall [35]. This highlights the importance of access to screening personnel across POC venues, as 4% of persons who tested positive and 19% of those who were negative reported that they were motivated by the convenience of time or location, and half of those who tested negative, along with 42% of positive persons, reported that they were tested as part of routine HIV status checks. Though anticipated, it is also noteworthy that referrals were a foremost testing motivator for persons who were positive, as one-third of those who received positive results reported that they had received a specific referral for HIV screening. Referrals from a healthcare facility (25%), friend, or family member (32%) accounted for more than half (57%) of all positive results, which may reflect promise for targeted campaigns aimed toward normalizing screening. Improving HIV status awareness may be particularly crucial for reducing risk in this population, as 21% of the HIV positive persons in this sample were unsure of their partner HIV status, more than half reported inconsistent condom use, and nearly a quarter of positive persons reported no condom use at all. 

 Primary limitations for this study included low representation of intravenous drug users and racial subgroups other than White and Black/African American. As an additional limitation was a reliance on the self-reported information that was gleaned via face-to-face testing and counseling interviews, low reporting of high risk drug behavior is not surprising. It is also important to note that, while 47% of individuals in the sample were tested through outreach, this yielded fewer positive results and confirmed linkages overall; a formal evaluation of outreach locations and processes will help to shape future efforts to respond to community requests for testing versus increasing targeted testing by area of demonstrated need. Outreach screening at regional health events may also have contributed to linkage challenges, as persons outside of the clinic service area may not have been eligible to link to care locally. Follow-up analyses for clients who did not attend a confirmed primary care visit and persons who were lost to follow up will provide supplementary insight on individual barriers to linkage. In addition, though this investigation utilized a convenience sample of test-seekers, this population was representative of the local urban community, and findings may be generalizable to other cities in the Southern Unites States.

 The overall linkage rate for positive persons in this sample was 86%, which included out-of-state residents, persons eligible for pediatric or obstetric care, persons who did not return for confirmatory results, individuals that were referred outside of Ryan White clinics, and persons who did not seek medical care locally, some of whom were lost to follow up despite staff contacts for up to 12 months after diagnosis. Significant differences in confirmed linkage were observed, specifically for non-White females, persons tested at outreach settings versus clinical settings, and for those who reported “convenience” as the motivation for testing. While we anticipated that some women may have elected to seek care with an existing gynecology or women's health provider/clinic, we did not predict that POC testing at outreach settings or test-seekers reporting being motivated by convenience would present significant unfavorable differences for confirmation of linkage in this cohort. Additional analyses on test-seeking clients who did not have a confirmed linkage to primary care are warranted, specifically to examine insurance status, clinical access, prior experience with the healthcare system, and novel mechanisms to minimize loss to follow up. 

 Challenges to linkage are not uncommon, and exploration of individual barriers to care, in combination with expanded integration of colocated linkage and surveillance efforts in the state or region, may improve linkage rates for marginalized, transient, and hard to reach individuals [31, 33, 50–52]. While further investigations may identify factors that keep some test-seekers from entering medical care following a positive diagnosis, these findings help to demonstrate the benefit of comprehensive POC testing and counseling and linkage services locally, as 97% of positive in-state test seekers who participated in an immediate linkage program (median days = 5) attended their first primary care visit (median days = 41). For persons who were successfully linked locally, 96% arrived for a first medical visit within 180 days of receiving a positive result, and 83% met the NHAS goal of linkage within 90 days [25], showing promise for integrated POC testing and linkage services.

## 4. Conclusions

Notable differences in race and gender, test location, and motivation for screening of persons who presented for POC HIV testing were observed for both test result and linkage to care in our investigation. Though rapid HIV, STI, and other POC testing innovations, including self- and in-home screenings, show promise for decreased diagnostic time with impressive results accuracy across locations [22, 31, 53–55], incorporating immediate active referrals and timely linkage to care services into these tactics is imperative for prompting early treatment initiation and improved outcomes [32, 56–58]. In order to fully realize the potential of sophisticated screening and surveillance technologies, it is essential to examine the dynamic population of HIV POC test-seekers in the Unites States. Enhancing programming for integrated, comprehensive HIV testing and linkage services for populations outside of primary healthcare settings should continue as a principal approach toward meeting the goals of the National HIV/AIDS Strategy and improving serostatus awareness, linkage rates, and HIV outcomes. 

## Figures and Tables

**Table 1:** Test results of clients who presented for HIV testing at clinic and outreach settings by year, *N* (%), 2007–2012.

Result	Test year						Total
	2007	2008	2009	2010	2011	2012	
Negative	684 (96)	723 (97)	572 (96)	459 (95)	533 (95)	532 (96)	3,503 (96)
Positive	25 (4)	26 (3)	26 (4)	23 (5)	26 (5)	22 (4)	148 (4)

Total	709	749	598	482	559	554	3,651

**Table 2:** Sociodemographic characteristics of clients who presented for HIV POC testing by test result, AL, 2007–2012.

Characteristic	Total	HIV positive	HIV negative	*P* value^a^
	*N* = 3,651	*N* = 148	*N* = 3,503	
	*n* (%)	*n* (%)	*n* (%)	
Age, years				0.05*
14 to 29	2,103 (58)	74 (50)	2,029 (58)	
≥30	1,535 (42)	74 (50)	1,461 (42)	
Sex				<0.001*
Male	1,756 (48)	116 (78)	1,640 (47)	
Female	1,887 (52)	32 (22)	1,855 (53)	
Race				0.03*
Black/AA	2,126 (59)	100 (68)	2026 (59)	
White	1,287 (36)	45 (31)	1242 (36)	
Other	171 (5)	2 (1)	169 (5)	
Race/gender				<0.001*
White female	535 (15)	4 (3)	531 (15)	
White male	750 (21)	41 (28)	709 (21)	
Non-white female	1,318 (37)	28 (19)	1290 (38)	
Non-White male	978 (27)	74 (50)	904 (26)	
Ethnicity				0.80
Hispanic	101 (3)	3 (2)	98 (3)	
Non-Hispanic	3,188 (97)	140 (98)	3,048 (97)	
Residence				0.84
Alabama	3,466 (96)	141 (96)	3,325 (96)	
Out of state	160 (4)	6 (4)	154 (4)	

*Statistically significant at 0.05 level.

AA: African American; HIV: Human Immunodeficiency Virus; POC: point of care.

Note: missing/unknown data (not shown) has been excluded for calculating percentages and *P* value.

^a^ *P* value (two tailed): Pearson chi-square test or Fisher's exact test.

**Table 3:** Reported characteristics of clients who presented for HIV POC testing by test result, AL, 2007–2012.

Characteristic	Total	HIV positive	HIV negative	*P* value^a^
	*N* = 3,651	*N* = 148	*N* = 3,503	
	*n* (%)	*n* (%)	*n* (%)	
*HIV testing *				
Test location				<0.001*
Outreach	1,723 (47)	8 (5)	1,715 (49)	
HIV clinic	1,896 (52)	140 (95)	1,756 (51)	
Previously tested				<0.001*
No	1,241 (34)	16 (11)	1,225 (35)	
Yes	2,378 (66)	131 (89)	2,247 (65)	
*Participant risk factors *				
History of STI^b^				<0.001*
Yes	491 (16)	36 (30)	455 (16)	
No	2,482 (83)	84 (70)	2,398 (84)	
Unsure	14 (1)	0 (0)	14 (<1)	
MSM^b^				<0.001*
Yes	550 (19)	70 (58)	480 (17)	
No	2,346 (80)	49 (41)	2,297 (82)	
Unsure	30 (1)	1 (1)	29 (1)	
History of illicit drug use^c^				0.26
Yes	642 (21)	31 (26)	611 (21)	
No	2,345 (79)	90 (74)	2,255 (79)	
Alcohol use				0.51
Yes	2,100 (70)	89 (73)	2,011 (70)	
No	889 (30)	33 (27)	856 (30)	
Condom utilization				0.001*
No condom use	889 (31)	29 (25)	860 (31)	
Condom use < 100%	1,276 (44)	71 (60)	1,205 (44)	
Condom use = 100%	719 (25)	18 (15)	701 (25)	

*Statistically significant at 0.05 level.

HIV: Human Immunodeficiency Virus; MSM: men having sex with men; POC: point of care; STI: sexually transmitted infections.

Note: missing/unknown data (not shown) has been excluded for calculating percentages and *P* value.

^a^ *P* value (two tailed): Pearson chi-square test or Fisher's exact test.

^b^“No” and “unsure” categories combined to calculate *P* value.

^c^Excluding marijuana.

**Table 4:** Reported participant and partner characteristics of clients who presented for HIV POC testing by test result, AL, 2007–2012.

Characteristic	Total	HIV positive	HIV negative	*P* value^a^
	*N* = 3,651	*N* = 148	*N* = 3,503	
	*n* (%)	*n* (%)	*n* (%)	
Number of sexual partners				0.01*
0 to 1	1,099 (37)	33 (27)	1,066 (38)	
2 to 9	1,563 (53)	67 (56)	1,496 (53)	
10 or more	286 (10)	20 (17)	266 (9)	
Partner HIV positive^b^				<0.001*
Yes	477 (14)	57 (40)	420 (13)	
No	2,379 (70)	56 (39)	2,323 (71)	
Unsure	561 (16)	30 (21)	531 (16)	
Partner is a clinic patient^b^				<0.001*
Yes	348 (12)	33 (26)	315 (11)	
No	2,495 (82)	83 (66)	2,412 (83)	
Unsure	186 (6)	10 (8)	176 (6)	
Partner is MSM^b^				<0.001*
Yes	566 (21)	68 (58)	498 (19)	
No	1,936 (71)	41 (35)	1,895 (73)	
Unsure	219 (8)	8 (7)	211 (8)	
Motivation for testing				
High risk event	358 (13)	16 (14)	342 (13)	0.76
Status/health update	1,412 (50)	50 (42)	1,362 (50)	0.10
Convenience/ease	527 (19)	5 (4)	522 (19)	<0.001*
Requirement for work/travel	118 (4)	4 (3)	114 (4)	1.00
Referred for testing	318 (11)	39 (33)	279 (10)	<0.001*
Other	99 (4)	4 (3)	95 (4)	1.00
Referral source				
Health professional	205 (7)	29 (25)	176 (7)	<0.001*
Public campaign/media	1,338 (48)	17 (15)	1,321 (49)	<0.001*
Self	238 (8)	5 (4)	233 (9)	0.10
Partner	267 (9)	20 (17)	247 (9)	0.004*
Friend/family member	617 (22)	38 (32)	579 (21)	0.005*
Other/nonspecific	153 (5)	8 (7)	145 (5)	0.49

*Statistically significant at 0.05 level.

Note: missing/unknown data (not shown) has been excluded for calculating percentages and *P* value.

HIV: Human Immunodeficiency Virus; MSM: men having sex with men; POC: point of care.

^a^ *P* value (two tailed): Pearson chi-square test or Fisher's exact test.

^b^“Unsure” category excluded for calculating *P* value.

**Table 5:** Sociodemographic characteristics of clients who presented for HIV POC testing by linkage, AL, 2007–2012.

Characteristic		Linked to care		*P* value^a^
	Total	Confirmed	Unknown/unconfirmed	
	*N* = 145	*N* = 124	*N* = 21	
	*n* (%)	*n* (%)	*n* (%)	
Age, years				0.54
14 to 29	74 (51)	62 (50)	12 (57)	
≥30	71 (49)	62 (50)	9 (43)	
Sex				0.02*
Male	113 (78)	101 (81)	12 (57)	
Female	32 (22)	23 (18)	9 (43)	
Race^b^				0.07
Black/AA	99 (69)	81 (66)	18 (86)	
White	43 (30)	40 (32)	3 (14)	
Other	2 (1)	2 (2)	0 (0)	
Race/gender				0.05*
White female	4 (3)	4 (3)	0 (0)	
White male	39 (27)	36 (29)	3 (14)	
Non-white female	28 (19)	19 (16)	9 (43)	
Non-white male	73 (51)	64 (52)	9 (43)	
Ethnicity				1.00
Hispanic	3 (2)	3 (3)	0 (0)	
Non-Hispanic	137 (98)	116 (97)	21 (100)	
Residence				1.00
Alabama	138 (96)	119 (96)	19 (95)	
Out of state	6 (4)	5 (4)	1 (5)	

*Statistically significant at 0.05 level.

AA: African American; HIV: Human Immunodeficiency Virus.

Note: Missing/unknown data (not shown) has been excluded for calculating percentages and *P* value.

^a^ *P* value (two tailed): Pearson chi-square test or Fisher's exact test.

^b^Categories “White” and “other” combined to calculate *P* value.

**Table 6:** Reported characteristics of clients who presented for HIV POC testing by linkage, AL, 2007–2012.

Characteristic		Linked to care		*P* value^a^
	Total	Confirmed	Unknown/unconfirmed	
	*N* = 145	*N* = 124	*N* = 21	
	*n* (%)	*n* (%)	*n* (%)	
*HIV testing *				
Test location				0.01*
Outreach	7 (5)	3 (2)	4 (19)	
HIV clinic	138 (95)	121 (98)	17 (81)	
Previously tested				0.47
No	16 (11)	15 (12)	1 (5)	
Yes	128 (89)	108 (88)	20 (95)	
*Participant risk factors *				
History of STI				0.39
Yes	36 (30)	29 (29)	7 (39)	
No	83 (70)	72 (71)	11 (61)	
MSM^b^				0.32
Yes	68 (58)	59 (60)	9 (47)	
No	49 (41)	39 (39)	10 (53)	
Unsure	1 (1)	1 (1)	0 (0)	
History of illicit drug use^c^				1.00
Yes	31 (26)	26 (26)	5 (26)	
No	88 (74)	74 (74)	14 (74)	
Alcohol use				0.60
Yes	88 (73)	75 (74)	13 (68)	
No	32 (27)	26 (26)	6 (32)	
Condom utilization				0.51
No condom use	28 (24)	26 (26)	2 (13)	
Condom use < 100%	71 (61)	59 (59)	12 (75)	
Condom use = 100%	17 (15)	15 (15)	2 (13)	

*Statistically significant at 0.05 level.

HIV: Human Immunodeficiency Virus; MSM: men having sex with men; POC: point of care; STI: sexually transmitted infections.

Note: missing/unknown data (not shown) has been excluded for calculating percentages and *P* value.

^a^ *P* value (two tailed): Pearson chi-square test or Fisher's exact test.

^b^“No” and “unsure” categories combined to calculate *P* value.

^c^Excluding marijuana.

**Table 7:** Reported participant and partner characteristics of clients who presented for HIV POC testing by linkage, AL, 2007–2012.

Characteristic		Linked to care		*P* value^a^
	Total	Confirmed	Unknown/unconfirmed	
	*N* = 145	*N* = 124	*N* = 21	
	*n* (%)	*n* (%)	*n* (%)	
Number of sexual partners				0.34
0 to 1	33 (28)	28 (28)	5 (28)	
2 to 9	66 (55)	58 (57)	8 (44)	
10 or more	20 (17)	15 (15)	5 (28)	
Partner HIV positive^b^				0.17
Yes	56 (40)	51 (43)	5 (25)	
No	56 (40)	46 (38)	10 (50)	
Unsure	28 (20)	23 (19)	5 (25)	
Partner is a clinic patient^b^				0.22
Yes	33 (27)	30 (28)	3 (16)	
No	82 (66)	67 (64)	15 (79)	
Unsure	9 (7)	8 (8)	1 (5)	
Partner is MSM^b^				0.79
Yes	66 (57)	56 (58)	10 (53)	
No	41 (36)	34 (36)	7 (37)	
Unsure	8 (7)	6 (6)	2 (10)	
Motivation for testing				
High risk event	16 (14)	14 (14)	2 (11)	1.00
Status/health update	49 (42)	41 (42)	8 (44)	0.84
Convenience/ease	5 (4)	2 (2)	3 (17)	0.03*
Requirement for work/travel	4 (3)	3 (3)	1 (6)	0.50
Referred for testing	39 (34)	37 (38)	2 (11)	0.03*
Other	3 (3)	1 (1)	2 (11)	0.06
Referral source				
Health professional	28 (24)	24 (25)	4 (22)	1.00
Public campaign/media	17 (15)	13 (13)	4 (22)	0.30
Self	5 (4)	4 (4)	1 (6)	0.58
Partner	19 (17)	17 (18)	2 (11)	0.73
Friend/family member	38 (33)	32 (33)	6 (33)	0.98
Other/nonspecific	8 (7)	7 (7)	1 (6)	1.00

*Statistically significant at 0.05 level.

Note: missing/unknown data (not shown) has been excluded for calculating percentages and *P* value.

HIV: Human Immunodeficiency Virus; MSM: men having sex with men; POC: point of care.

^a^ *P* value (two tailed): Pearson chi-square test or Fisher's exact test.

^b^“Unsure” category excluded for calculating *P* value.
